# Predicting brain age across the adult lifespan with spontaneous oscillations and functional coupling in resting brain networks captured with magnetoencephalography

**DOI:** 10.1162/imag_a_00195

**Published:** 2024-06-17

**Authors:** Samuel Hardy, Gill Roberts, Matthew Ventresca, Benjamin T. Dunkley

**Affiliations:** MYndspan Ltd, London, United Kingdom; Neurosciences and Mental Health, Hospital for Sick Children Research Institute, Toronto, Canada; Diagnostic and Interventional Radiology, Hospital for Sick Children, Toronto, Canada; Medical Imaging, Faculty of Medicine, University of Toronto, Toronto, Canada

**Keywords:** magnetoencephalography, brain ageing, healthy ageing, functional connectivity, neural oscillations, resting-state networks, intrinsic brain networks, brain age prediction

## Abstract

The functional repertoire of the human brain changes dramatically throughout the developmental trajectories of early life and even all the way throughout the adult lifespan into older age. Capturing this arc is important to understand healthy brain ageing, and conversely, how injury and diseased states can lead to accelerated brain ageing. Regression modelling using lifespan imaging data can reliably predict an individual’s brain age based on expected arcs of ageing. One feature of brain function that is important in this respect, and understudied to date, is neural oscillations—the rhythmic fluctuations of brain activity that index neural cell assemblies and their functioning, as well as coordinating information flow around networks. Here, we analysed resting-state magnetoencephalography (MEG) recordings from 367 healthy participants aged 18 to 83, using two distinct statistical approaches to link neural oscillations and functional coupling with that of healthy ageing. Spectral power and leakage-corrected amplitude envelope correlations were calculated for each canonical frequency band from delta through gamma ranges. Spatially and spectrally consistent associations between healthy ageing and neurophysiological features were found across the applied methods, showing differential effects on neural oscillations, with decreasing amplitude of low frequencies throughout the adult lifespan, and increasing high-frequency amplitude. Functional connectivity within and between resting-state brain networks mediated by alpha coupling generally decreased throughout adulthood and increased in the beta band. Predictive modelling of brain age via regression showed an age-dependent prediction bias, resulting in overestimating the age of younger people (<40 years old) and underestimating the age of older individuals. These findings evidence strong age-related neurophysiological changes in oscillatory activity and functional networks of the brain as measured by resting-state MEG and that cortical oscillations are moderately reliable markers for predictive modelling. For researchers in the field of predictive brain age modelling with neurophysiological data, we recommend attention is paid to predictive biases for younger and older age ranges and consider using specific models for different age brackets. Nevertheless, these results suggest brain age prediction from MEG data can be used to model arcs of ageing throughout the adult lifespan and predict accelerated ageing in pathological brain states.

## 1 Introduction

Brain activity changes throughout the lifespan, rapidly in the early life developmental arcs during brain maturation (Hunt et al., 2019;Manza et al., 2015), through early-middle adulthood, and into older age (Vlahou et al., 2014). Neural oscillations offer a window in brain development, maturation, and ageing (Gómez et al., 2013), capturing neurophysiological processes at the population level (Rempe et al., 2023). Oscillatory activity can be captured via electroencephalography (Srinivasan et al., 2006) and magnetoencephalography (MEG). MEG is a functional neuroimaging technique that records the neuromagnetic activity of the brain via an array of magnetometers/gradiometers. Resting-state MEG is used to assess the spontaneous and intrinsic neural activity of individuals not engaged in an explicit task, revealing neurophysiological activity with minimal cognitive load. The benefit of MEG compared to other functional neuroimaging modalities such as functional magnetic resonance imaging (fMRI) and EEG is the greater temporal and spatial resolution, respectively, facilitating collection of “richer” datasets in equal time, while remaining completely non-invasive despite directly recording neural activity. Resting-state MEG has been shown to be sensitive to age-related changes in neurophysiological activity (Hunt et al., 2019;Rempe et al., 2023;Vlahou et al., 2014), as well as a biomarker source for a diverse set of neurological and psychiatric states (Allen et al., 2021;Dunkley et al., 2014;Huang et al., 2014;O’Reilly et al., 2017), and can even differentiate between disorders with significant symptom overlap (Zhang et al., 2021).

Age is a primary risk factor for most neurodegenerative diseases, and MEG has been leveraged in studies of neurodegeneration such as Alzheimer’s and other dementias (Engels et al., 2016;Wiesman et al., 2022) and Parkinson’s (Vardy et al., 2011). Characterising the typical course of healthy ageing in the adult human brain will help us to understand the importance of deviation from expected trajectories of age-dependent neurophysiological features, and if such deviation is associated with neurodegeneration and accelerated neural ageing (Ye et al., 2020). Functional neuroimaging derived features would define new pathways for the early detection of pathological brain states, beneficial in cases where pre-symptomatic pathology is most effectively treated with early intervention.

Neural oscillations are a key feature of neural processing and reflect rhythmic activity of neural assemblies, across multiple spatial and temporal scales. Importantly, they have been implicated in information processing and transfer, perceptual binding, cognitive processes, and behavioural output. Furthermore, they provide mechanistic information about disease state (Mathalon & Sohal, 2015). Oscillatory activity can be recovered from neural spectral power, and is a biophysical phenomenon used to describe the activity of frequency specific processing throughout the brain. Associations between healthy ageing and M/EEG detected neural activity generally detail a profile of consistent spectral changes, including oscillatory activity shifting from low to high frequencies, as measured via band limited power (Barry & De Blasio, 2017) and full power spectra (Merkin et al., 2023;Voytek et al., 2015). These spectral changes are not spatially uniform across the cerebral cortex, evidenced by theta and alpha showing the strongest decreases in the posterior regions, and conversely beta activity being most acutely increased across the frontal hemispheres (Barry & De Blasio, 2017).

Neural oscillations also mediate functional connectivity (FC), and in electrophysiological contexts, FC is usually defined by phase- or amplitude-based coupling modes (Engel et al., 2013). Amplitude envelope correlations (AEC), however, are generally the most stable (Colclough et al., 2016) and reliable over time (Garcés et al., 2016), and intrinsic resting networks recovered from AEC closely resemble those recovered by other imaging modalities, such as more widely used fMRI (Brookes, Hale, et al., 2011). Studies of resting-state FC using fMRI have demonstrated age-related changes in the amygdala (Xiao et al., 2018) and basal ganglia (Manza et al., 2015) between young and middle adulthood, as well as in the default mode network (Ferreira & Busatto, 2013). While fMRI measures neural activity indirectly via blood-oxygen-level-dependent (BOLD) signals with a temporal resolution of approximately 1–5 seconds, MEG directly measures the neuromagnetic activity of the brain with millisecond resolution, allowing connectivity to be measured without the limitation of the haemodynamic response time. Nevertheless, there is excellent spatial and spectral congruence between MEG and fMRI measures of resting-state FC (Brookes, Hale, et al., 2011;Brookes, Woolrich, et al., 2011), and MEG-based studies have revealed differences in network topology between children, adolescents, and early-middle adulthood (Schäfer et al, 2014).

The relationships between neural activity and age are often improperly modelled as purely linear; however, the true trajectories of these features with age appear to be generally monotonic, though quadratic relationships are also present (Gómez et al., 2013;Hunt et al., 2019) but are weak enough to have most variation captured by a monotonic fit. Additionally, research in this area is often limited by mixed modality use (Voytek et al., 2015), limited sample sizes (Barry & De Blasio, 2017;Voytek et al., 2015), narrow age ranges (Hunt et al., 2019;Schäfer et al, 2014), and group-wise rather than continuous analysis of ageing (Koelewijn et al., 2017;Merkin et al., 2023), which highlights the need for further analysis of large M/EEG datasets in this area which comprehensively cover the typical adult human lifespan in a continuous manner.

Using neuroimaging modalities to predict age is an established technique, commonly referred to as “brain age” (Cole et al., 2019). This process involves modelling the effect of age on neuroimaging data as collected from many samples to account for personal variation and limit bias. As variation is present in functional and structural brain data within even limited age groups, predictions (i.e., brain age) frequently differ from chronological age, with the difference being called residuals or brain age delta (BD). Since BD is found by subtracting the real value of age from brain age, a positive BD means a predicted age higher than chronological age, which is a result of brain data which more closely resembles data from an individual older than chronological age would imply. Given the potential for difference between chronological and brain age, research has been conducted to explore if this residual error is related to biomarkers of various pathologies, primarily but not limited to those of a neurological nature (Lee et al., 2022;Rauseo et al., 2023;Ye et al., 2020).

This work will explore age-related effects on neurophysiological features, including spontaneous cortical activity and FC derived from resting-state MEG data. We replicate prior findings on cortical oscillatory and network activity, and advance the field significantly by revealing the age-related changes from early-to-late adulthood in intrinsic functional networks, a novel approach with MEG data. In doing this, we also determined stable features of maturation across the adult lifespan, allowing comparison to existing literature in this subject area. Additionally, we performed regression analysis to predict chronological age using MEG-derived biophysical features of brain activity, and show that brain age predictive modelling using cortical oscillatory activity on the whole is moderately reliable, but that there are small age-related biases in predictions that researchers using neurophysiological data should be mindful of.

## 2 Methods

### 2.1 Participants

Participants’ data were compiled from four distinct cohorts and sites, including The Hospital for Sick Children Toronto (SickKids) (n = 18), The Open MEG Archive (OMEGA) healthy controls (n = 106), National Institute of Mental Health (NIMH) healthy research volunteers (n = 49), and a subset of the Donders Institute Mother of Unification Studies (MOUS) (n = 194). For each participant, we utilised T1 MRI for source-localisation and eyes-open resting-state MEG neuroimaging data acquired from a CTF-275 MEG system (CTF MEG Neuro Innovations Inc. Coquitlam, BC, Canada) with the exception of the SickKids dataset, which was recorded using a CTF-151 system from the same manufacturer. At least 5 minutes of resting-state data was acquired for each participant. The total cohort size is 367 participants (mean age = 36.51 ± 20.07 years, age range = 18–83 years, female = 59.94%), with the age distribution of each distinct cohort shown inFigure 1. All data were collected under ethical approval from each respective site’s Research Ethics Board / Institutional Review Board (REB/IRB), and all participants provided informed consent to participate in research.

**Fig. 1.**
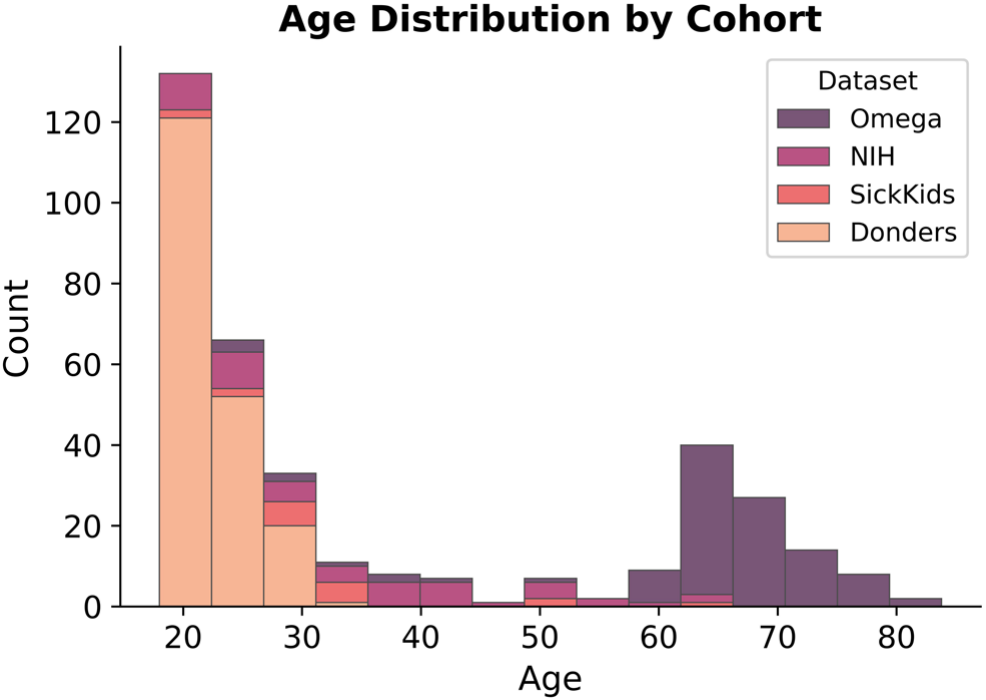
Histogram of participant age, grouped by cohort.

### 2.2 MEG data and processing

#### 2.2.1 Pre-processing

All MEG data were processed with a custom pipeline utilising the open source MNE-Python software package (Gramfort et al., 2013). CTF MEG systems use a reference array of magnetometers to record magnetic fields distant from the head simultaneously with neuromagnetic fields. This enables the interfering fields present in the room to be measured and attenuated in software using a synthetic third-order gradiometry scheme; a detailed explanation of this setup can be found inVrba and Robinson (2001). We used this method to perform interference reduction on all MEG data used in our analysis. Recordings were temporally cropped to the first 5 minutes of the recording, then were band pass filtered using a zero-phase FIR filter (impulse response duration 3.3 seconds) between 1 and 150 Hz, and resampled to a frequency of 300 Hz before processing. A notch filter was used to remove line noise at 50 and 60 Hz. Subsequently, independent components analysis was performed with 80 components identified for each scan using the infomax approach (Lee et al., 1999). Artefactual components (ocular, cardiac) were identified automatically using a proprietary machine learning classifier and excluded.

Lastly, the sensor-level data were segmented into non-overlapping epochs of 10.24 seconds each. Any epochs with a peak-to-peak amplitude greater than 6000 fT were discarded from further analysis; any epochs with high-frequency (>110 Hz) activity greater than 2000 fT were also discarded, a threshold which was chosen to reject epochs containing high-frequency muscle activity. The remaining clean epochs were discarded until 250 seconds of data were available, ensuring that the same length of data was analysed for all scans.

#### 2.2.2 Forward modelling

We used a 1-shell boundary element model, with the geometry warped to fit each subject’s anatomical MRI, to calculate magnetic lead fields (Nolte, 2003); these lead fields were subsequently used in the inverse solution for neural source reconstruction. Specifically, the subject’s T1 MRI was resampled to isotropic 1 mm resolution and underwent an affine (non-rigid) registration to a template brain using Freesurfer software (Fischl, 2012). This registration defines a correspondence between individual subject source spaces and the template space. The template includes precomputed boundary meshes defining skull compartments; we transformed the inner skull mesh, along with source locations of interest, back to each subject-specific source space.

Dipolar sources were modelled at 122 locations across the brain. These fall into two categories: 78 locations from the AAL atlas (Gong et al., 2009) which were used for regional power estimates, and 44 other MNI coordinates which were used as network nodes (Network Nodes Definitions inSupplementary Materials). These locations were selected a-priori to model five specific large-scale networks: the central executive (CEN), default mode (DMN), motor (MOT), visual (VIS), and attention (ATT) networks. The specific MNI coordinates were adapted from previous work in the field (De Pasquale et al., 2012;Gong et al., 2009;Hsu et al., 2014); specific node groupings may be found in theSupplementary Materials.

#### 2.2.3 Source imaging

LCMV (linearly constrained minimum variance) beamforming is a spatial filtering method which can be applied to sensor-level MEG data to compute linear combinations of sensor channels which estimate source-level activity (Van Veen et al., 1997). The LCMV weights are chosen to minimise overall signal variance for a given location subject to the constraint of unit gain, which enforces that signals originating from the chosen location must be passed by the filter.

Multi-channel MEG sensor data can be represented as anm×ndata matrix**B**, wheremis the number of independent channels andnis the number of samples in time. We also have a forward model which provides the lead fields at those channelsH(m×1)for a given current dipole amplitudeq(r,θ)with orientationθat positionr. This forward model provides the unit gain constraint for the beamformer weightsW:

$${\text{W}}^{T}\text{H}=\text{I}$$ (1)

The beamformer weights vectorwis designed such that estimated source activity$\hat{q}\left(t\right)$is a linear combination of the weights and the data matrix, that is

$$\hat{q}(\text{r},\theta ,t)=\text{W}(\text{r},\theta )\text{B}(t)$$ (2)

While the derivation is beyond the scope of this work, the optimal weights have a closed-form expression in terms of the data covarianceCand lead fieldsH:

$$\text{W}(\text{r},\theta )=\frac{{\text{H}}^{T}{\text{C}}^{-1}}{{\text{H}}^{T}{\text{C}}^{-1}\;\text{H}}$$ (3)

The estimated variance at a particular location contains a spatially inhomogeneous noise component, which increases towards the regions of lowest signal in the source space (typically near the centre of the head). Since we are recording in a “contrast free” resting-state condition, we assume noise is uncorrelated across seconds and normalise the source estimates by${\text{H}}^{T}\text{H}$such that we obtain the quantity called the “neural activity index” byVan Veen et al. (1997).

For each subject, cleaned sensor-level epochs were first concatenated in time and used to compute the covariance matrix. We applied Tikhonov regularisation to the covariance matrix to increase numerical stability of the inversion, using 5% of the maximum singular value applied to the diagonal elements of the matrix. Epochs were spatially filtered at the 122 source locations using three independent cartesian components for each source; these were reduced to scalar source time courses (STCs) using a singular value decomposition method (Sekihara et al., 2004) to determine the orientation of maximum power. The STCs were then independently scaled to unit standard deviation.

We estimated power spectral density from concatenated STCs for each of the 78 AAL locations using Welch’s method, computed using non-overlapping segments of 4096 samples for each FFT and then averaged. This provides a stable estimate of the relative frequency contribution of power at each virtual electrode. The resulting power spectra were divided into five a-priori frequency bands (seeTable 1) with a 1 Hz gap to remove overlap, and the mean amplitude for each frequency band was computed; this results in five estimates of band-limited relative power at each AAL node.

**Table 1.** Frequency band definitions.

Band	Delta	Theta	Alpha	Beta	Gamma
Frequency (Hz)	1-3	4-8	9-12	13-29	30-45
IR duration (seconds)	3.3	1.65	1.65	1.015	0.44

Impulse response durations are also included.

Source time courses from the network nodes were used to estimate connectivity. Firstly, the time courses were band pass filtered using a zero-phase FIR filter for each frequency band (seeTable 1for impulse response duration), then orthogonalised using an iterative symmetric procedure to remove zero-lag correlations; this removes any spurious correlations which would otherwise result from magnetic field spread (Colclough et al., 2015). We then used the Hilbert transform to estimate the amplitude envelope for each STC. These envelopes were downsampled to 1 Hz and the Pearson correlation coefficientρbetween pairs of nodes was calculated, following a procedure outlined inBrookes, Hale, et al. (2011). The output takes the form of an NxN matrix, where each element defines the correlation between a pair of nodes.

### 2.3 Partial least squares

Partial least squares (PLS) is a cross-decomposition method similar to principal component analysis (PCA), but whereas PCA reduces the dimensionality of a matrix such that the resulting components maximally explain the variance of the matrix (while remaining orthogonal), PLS instead produces two sets of components, between pairs of which the covariance is maximised (Abdi & Williams, 2013). PLS is useful in high-dimensional data in which dimensionality reduction with respect to a specific set of targets is ideal, and further where the regressors may be multi-collinear. We used PLS to explore the relationships between MEG data and metadata, such as age and sex, as a means of further exploring how these variables are represented neurophysiologically. This analysis is exploratory in nature, and performed using the entire dataset, it is used to establish any population-level relationship with age and is completely distinct to the subsequent regression analysis. To perform PLS, we begin with the calculation of the cross-product matrix as shown inequation 4, in which**X**is the independent variables (MEG features), and**Y**is the dependent variables (metadata), where**X**and**Y**have both been centred and normalised.

$$R=Y^{T}X$$ (4)

The singular value decomposition (SVD) of**R**is then calculated usingequation 5, where**U**and**V**are the left and right singular vectors respectively, and Δ is the singular values. Right and left singular vectors represent the metadata and MEG features respectively which best describe**R**.

$$R=U\Delta V^{T}$$ (5)

Components (also called latent variables) are then produced via projection of the MEG features onto the left singular vector (**V**), and the metadata onto the right singular vector (**U**) as shown inequations 6and7.

$$L_{X}=XV$$ (6)

$$L_{Y}=YU$$ (7)

These latent variables are often called scores, and pairs of scores such as**Lx_0_**and**Ly_0_**are designed to be maximally covariant and represent the relationship between the**X**and**Y**features to the extent that the common information in the two matrices allows.

To test the significance of the produced latent variables, we compare the singular values (Δ ofequation 5) of the SVD of**X**and**Y**to null distributions of the singular values generated by permutation testing. This process involves shuffling**X**and recomputing the singular values via a new SVD under the assumption of no relationship between**Y**and shuffled**X**. For each singular value of the original SVD, we can use the corresponding null distribution to calculate the*p*-value which describes the probability of witnessing a singular value at least as extreme as observed. It is possible for only a subset of the singular values of an SVD to be found statistically significant (α = 0.05), and in this case only those which are significant are considered for further analysis.

In assessing the relationship between the latent variables of**Y**(**Ly**) and the features (columns) of**Y,**we first used a spearman’s rank correlation. Furthermore, we produced confidence intervals of these correlations via bootstrapping, whereby 1000 resamples with replacement are performed, and the SVD and subsequently the latent variables are computed for each, with the correlation coefficients between these latent variables and the bootstrapped**Y**features being used to generate confidence intervals.

### 2.4 Regression

For all modelling, the regressor variables of MEG-derived spectral power and/or FC features are used to predict the continuous response variable of chronological age.

Sci-kit Learn (Pedregosa et al., 2011) is used for its cross-validation, standardisation, and modelling functionality in this study. To evaluate the performance of each model, we used repeated K-Fold (k = 10) cross-validation, in which 10 repetitions are carried out. Within each cross-validated test, the model input (MEG) features are standardised (using the training folds) to have a mean of zero and a standard deviation (SD) of one. Additionally, we stratify the response variable of age into 4 linearly spaced groups (using the limits 0.0, 22.5, 45.0, 67.5, 90.0), to ensure that each fold contained an adequate age distribution. Response variable stratification is used to limit the effect of skewed response variable distributions in training folds limiting the generalisation of models. Although stratification is performed over grouped ages, prediction is performed against the continuous ungrouped value of chronological age. Furthermore, the use of additional cross-validation folds could prove insightful; however, our dataset has limited coverage of the 40–60 age range and increasing the number of folds would strain the ability of each fold to contain a stratified sample. The performance of all models is measured using mean absolute error and r-squared, with the mean and SD of these metrics for training and testing data over cross-validation being recorded.

Regression models were selected which have previously shown promise in similar domains and tasks, particularly regularised linear models, ensemble tree models, support vector machines, and multilayered perceptron models. These methods represent a diversity of architecture that is designed to ensure a particular regression approach does not limit the scope of achievable performance. When establishing the hyperparameter space of each model, between two to five sets of values were typically selected for each hyperparameter. The scale of these values was determined based on their impact on the model, for instance, regularisation favours values selected at log scale, whereas hyperparameters referencing number of samples or features were selected at linear scale. Additionally, both linear and non-linear kernels were evaluated where possible.

To tune the hyperparameters of each of the tested regression models, we performed a cross-validated grid search over each model’s hyperparameter space, using the same cross-validation procedure previously specified. This analysis was replicated using varying input features, with the performance of each subsequent set of models informing the next test. Initially, the input features were spectral power and FC, followed by each of these features independently, and finally principal component analysis (PCA) using spectral power, and spectral power with FC. The inclusion of FC as a model input proved detrimental to model performance, which predicated the use of dimensionality reduction to reduce collinearity across FC values and furthermore limit the impact of any noise at an attribute level. However, inclusion of FC with spectral power failed to improve model performance compared to PCA using only spectral power, therefore it was not tested individually with PCA.

For each model architecture we selected the best performing parameterisation to compare against alternative architectures and input features. Based on the results of this analysis, we then selected the two best performing models (RidgeCV and LassoCV), which exhibited distinctly higher performance than the other models. We then explored if additional performance could be extracted via a meta-estimator (AdaBoostRegressor). This process necessitated hyperparameter tuning of the meta-estimator independently for each selected model, which was performed using the same cross-validated grid search strategy previously employed.

Existing literature details at length a common pitfall of predicting age using neuroimaging features as prediction bias, particularly over-predicting (positive residuals) for younger subjects, and under-predicting (negative residuals) for older subjects. Multiple bias correction methods exist which attempt to address this issue (Peng et al., 2021;de Lange & Cole, 2020), and following the reasoning specified we chose to train a regression model (on the train folds) using chronological age to predict the age prediction of the main model. This process is used to find the line of best fit between the training labels and training predictions; the equation of that line is then rearranged such that we can transform the training predictions to be distributed around the ideal regression line (where the slope is 1). Detailed inequation 8is the equation of the line for predictions against chronological age, where**z**,**c**,**a**, and**b**are brain age, chronological age, the slope, and the intercept respectively. The training and testing data are then bias corrected by subtracting the intercept from the main model predictions and dividing by the slope as shown inequation 9, where**z`**is the resulting bias-corrected brain age.

z=ac+b (8)

$$z`=\frac{z-b}{a}$$ (9)

This approach ensured no data-leakage in the bias-correction process; however, it does assume that the bias of the training data predictions is approximately equal to that of the test data predictions. Given that the difference in the bias between training and testing predictions is a proxy for over/underfitting, the benefit afforded by the bias correction is greatest when the difference between training and testing performance metrics is small. This is assessed after a model is trained, to determine if a model will benefit from bias correction. Furthermore, this process does not impact the training of the brain age model in any way. A diagram of the full analysis methodology can be found in Analysis Methodology ofSupplementary Materials.

## 3 Results

### 3.1 PLS reveals cortical oscillatory and connectomic changes associated with healthy ageing

**Fig. 2.**
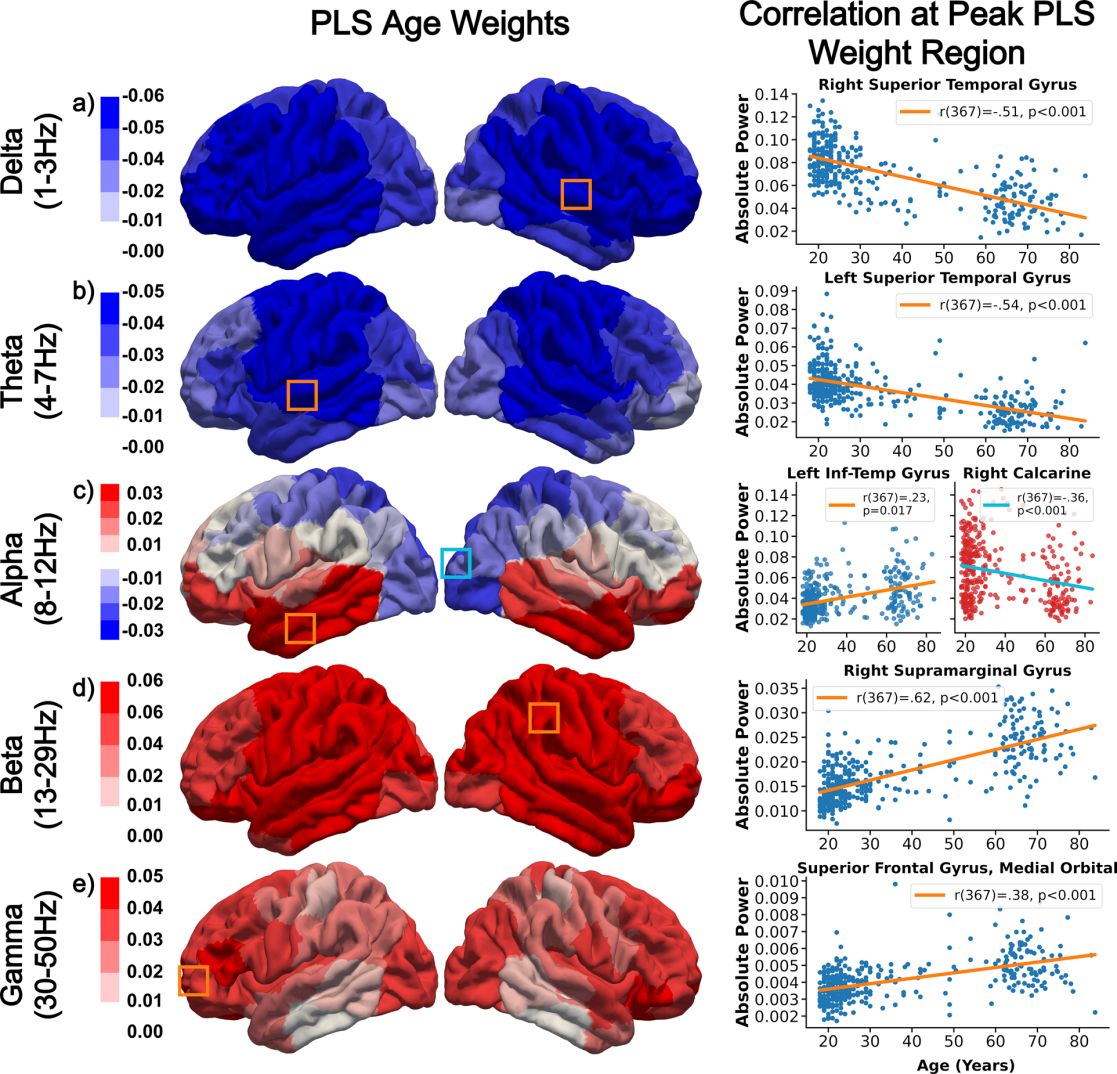
Spectrally and spatially specific effects of ageing on cortical oscillatory activity. Low-frequency (e.g., delta and theta) decreases in power with age are prominent in frontal and temporal regions, with decreases in alpha seen in the occipital regions. Age associations in alpha are diminished with respect to the spectrally adjacent theta and beta bands, which exhibit stronger and exclusively unidirectional changes. The most pronounced age-related changes in power are in beta, in which increasing activity across all brain regions is associated with healthy ageing. Similarly, gamma activity was found to diffusely increase with ageing, primarily in frontal regions. Labels a-e highlight band specific coefficients of healthy ageing for delta, theta, alpha, beta, and gamma respectively. Bounding boxes on the band limited power plots highlight PLS coefficient peak regions; the colour of the bounding box corresponds to the line of best fit shown on the adjacent scatter plot, which shows data from this region.

Cortical oscillatory activity shows strong age-dependent effects, with low frequencies, including delta and theta, showing global decreases across the adult life span (Fig. 2a, b), while high frequencies, such as beta and gamma, show widespread increases (Fig. 2d, e), with alpha showing spatially specific differential relations. Additionally, the coefficients of spectral power shown inFigure 2present the same general trends with respect to age as the partial correlation coefficients (Fig. S2in Supplementary Materials), specifically, decreasing delta activity except in the occipital lobe, decreasing theta focussed in the temporal and parietal regions, decreasing occipital alpha and increasing temporal alpha, increasing global beta power, and increasing gamma across the whole brain but prominently in frontal areas and most weakly in temporal regions. Generally, the effect of healthy ageing found here is a shift toward high-frequency activity, with the alpha band capturing the pivot point around which activity shifts, evidenced by its status as the only frequency band exhibiting bi-directional changes associated with healthy ageing.

The following regions have the largest frequency band specific absolute coefficient value in PLS analysis for predicting age, right superior temporal gyrus (***r***(367) = -.51,***p***= 6.24e-20), left superior temporal gyrus (***r***(367) = -.54,***p***= 1.30e-20), right calcarine (***r***(367) = -.36,***p***= 4.48e-9), right supramarginal gyrus (***r***(367) = .62,***p***= 1.43e-27), and left superior frontal gyrus, medial orbital (***r***(367) = .38,***p***= 1.08e-9) respectively from the previously mentioned canonical frequency bands.

**Fig. 3.**
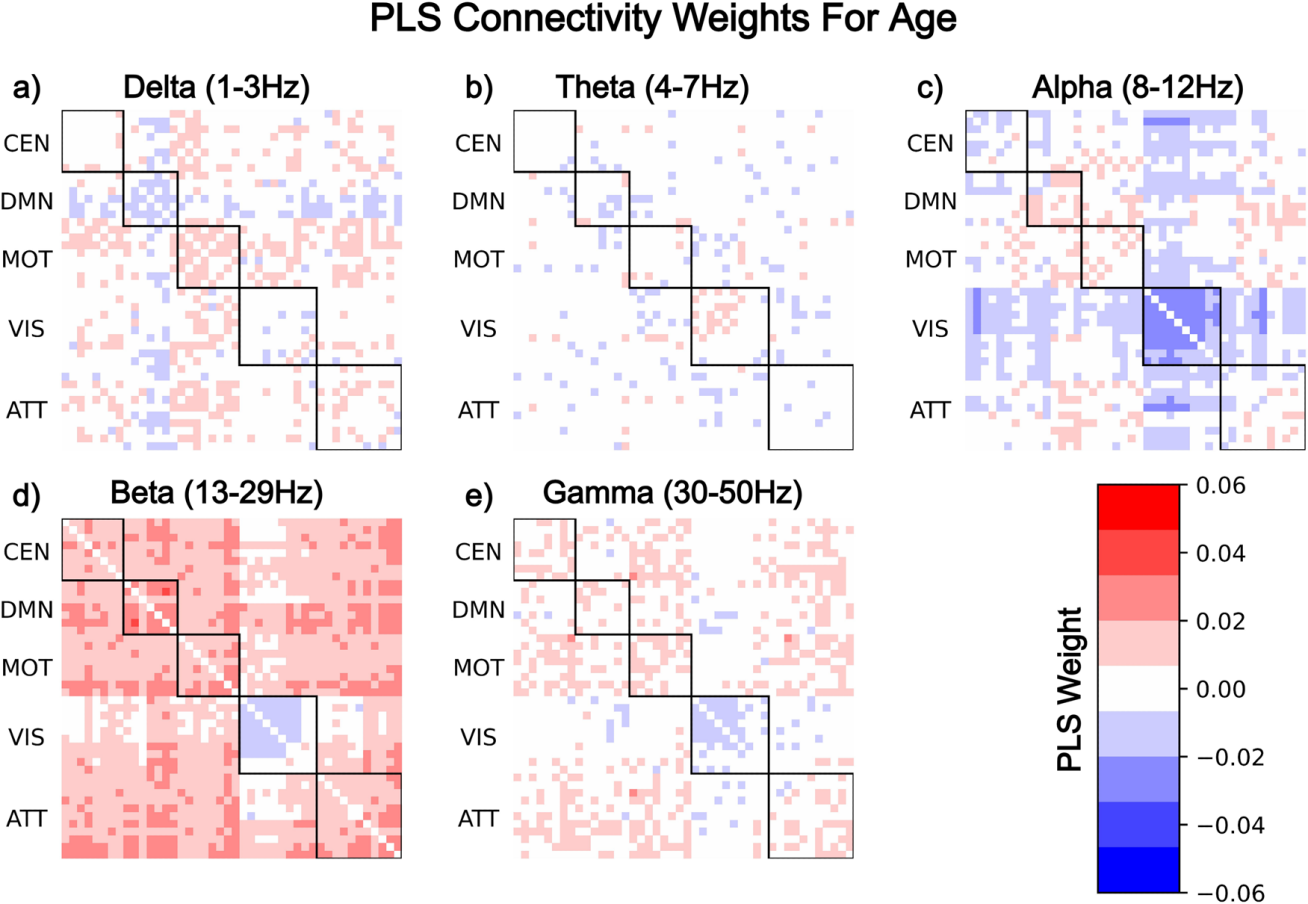
Functional connectivity shows network- and frequency-specific changes associated with healthy ageing, with inter- and intra-default mode network connectivity decreasing in delta, while theta exhibits no clear effect beyond single node connectivity changes. Connectivity is decreased within the visual network across alpha, beta, and gamma, with the effect most exaggerated in alpha, where inter visual network connectivity also decreases to a lesser extent. Outside of the visual network, beta and gamma demonstrate diffuse increasing connectivity associated with healthy ageing. Labels a-e refer to coefficients of healthy ageing for delta, theta, alpha, beta, and gamma frequency bands respectively.

Healthy ageing was found to be associated with multiple network-level features of FC, including decreases in the default mode network in delta (Fig. 3a), decreases in the visual network in alpha, beta, and gamma (Fig. 3c, d, e), as well as diffuse connectome wide increases in beta and gamma (Fig. 3, e). The average absolute PLS coefficient value of connectivity is significantly lower than that of the spectral power coefficients (***U***= 107464,***p***= 9.33e-186), highlighting that spectral power features have greater association with age on average. This is also reflected inFigure 3which shows the comparatively low coefficient values for connectivity. Furthermore, these results show region, frequency band, and directional concordance with partial correlation coefficients of the default mode of beta, as well as the visual network in alpha, beta, and gamma (Fig. S3in Supplementary Materials).

### 3.2 MEG-derived oscillatory activity for predicting functional brain age

A variety of models provided in the sci-kit learn python package were evaluated via hyperparameter tuning in the model selection process; the best model which is subsequently used in the following sections is an adaboosted (Drucker, 1997) cross-validated ridge regression model parameterised to have strong regularisation, and only use spectral power features. This particular model was selected as it produced the lowest summed value of mean test fold performance and the difference between train and test fold performances (as measured via mean absolute error). Using the difference between train and test performance metrics is intended to penalise overfit models which would not work well with the specified bias correction procedure. Various subsets of the features were used to test the models, as well as decomposition methods such as PCA, with results showing that using PCA and only spectral power both independently reduced overfitting, though PCA also worsened test fold performance.

#### 3.2.1 Raw predictions

As expected, a degree of bias exists in the age predictions, as visible inFigure 4and the performance metrics listed inTable 2. It is also clear that the severity of the bias is increased in the test set compared to the training data, which, while anticipated, does limit the effectiveness of the bias corrected procedure later used, as the degree of difference is substantial. The prediction bias found follows the trend of existing literature in this domain, in which younger people’s ages are overpredicted (positive average brain age delta), and older individuals’ ages are underpredicted (negative average brain age delta). The use of age stratified cross-validation does appear to have limited the detrimental effects of the data sparsity for the middle-aged subjects, with test fold performance not suffering in this age range compared to others.

**Fig. 4.**
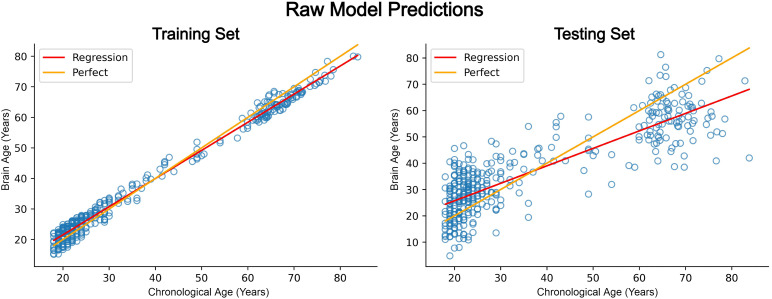
Model predictions show significantly reduced error over training samples compared to test set (unseen) samples, highlighting the presence of overfitting. Additionally, both training and testing predictions display age-dependent prediction bias, resulting in overestimating the age of younger people (<40 years old) and underestimating the age of older individuals (>40 years old), which is highlighted by the divergence of the ideal and actual lines of best fit of the predicted and actual values of age. The displayed data points represent intra-individual mean prediction across cross-validation folds within respective train and test splits.

**Table 2.** The regression model demonstrates overfitting to the training samples, evidenced by the disparity between the performance measures of the train and test sets.

	Mean absolute error	R ^2^
Train fold performance (mean ± SD)	2.38 ± .02	.98 ± 2.92e-4
Test fold performance (mean ± SD)	8.57 ± .06	.71 ± 3.5e-3

The test samples are predicted with mean absolute error of 8.57 years on average over cross-validation splits.

#### 3.2.2 Bias-corrected predictions

During each cross-validation iteration, the training fold predictions and labels are used to calculate the bias correction linear model; this model is then used to adjust the test fold data, which are then stored in both raw and bias-corrected form for each individual in the test fold. After all cross-validation iterations have been completed, the raw and bias-corrected test fold predictions are averaged for each individual, creating the results shown inFigures 4and5. As the bias correction models are calculated using the training folds to avoid data leakage, the bias of the test folds will only ever be adjusted to the degree that the bias is found in the train folds. If therefore the bias between these two data sets is substantially different, the bias of the test data will not be optimal, as seen inFigure 5, the right side of which highlights the amount of bias remaining (the degree of divergence between the regression and perfect fits) in the test data. Although in this case the bias correction is nonoptimal, it still demonstrates limited effectiveness (seeTables 2and3) as predictions are adjusted in the correct directions across the age continuum, but not to the ideal degree.

**Fig. 5.**
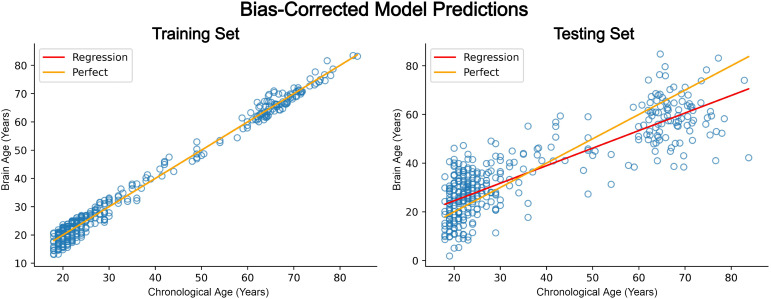
The effect of the bias correction on the training set is clear, as the divergence between ideal and actual fits is removed; however, the effect on the test samples is limited (due to overfitting), which is clear by the persistence of non-overlapping lines of best fit for ideal and actual test set predictions. Within-participant mean predictions are used in the plots above, with means being limited to the distinct train and test fold predictions for each participant.

**Table 3.** The performance values of the bias-corrected predictions are improved over the raw predictions, most prominently in the training performance, and to a lesser extent in the mean absolute error of test set performance, as shown via the mean and SD of the cross-validated performance metrics.

	Mean absolute error	R ^2^
Train fold performance (mean ± SD)	1.91 ± .02	.98 ± 3.35e-4
Test fold performance (mean ± SD)	8.54 ± .07	.71 ± 3.82e-3

## 4 Discussion

### 4.1 Summary

Neurophysiological activity develops and matures throughout the process of healthy ageing, reflecting underlying changes to cellular and population functioning. We sought to replicate prior work on the maturational effects of regional oscillatory activity, advance our understanding of oscillatory-mediated functional connectivity across resting-state brain networks, and test brain age prediction models on neurophysiological data. We replicated prior studies that found age-related decreases in low-frequency activity (Barry & De Blasio, 2017;Hunt et al., 2019), while simultaneously high-frequency activity (Barry & De Blasio, 2017;Gómez et al., 2013) tends to increase. Furthermore, we present novel results that show intrinsic coupling in key resting-state networks, including the DMN, CEN, and attention network, including age-related decoupling mediated by alpha activity, especially within and between the visual network, and across network increases in coupling mediated by beta activity. Finally, we present a regression model that tested brain age prediction modelling on neurophysiological data and found that the age of younger individuals tends to be overpredicted and the age of older individuals is underpredicted, calling attention to the sensitivity of the tested bias-correction procedure to overfitting.

### 4.2 Oscillatory activity throughout adulthood

Oscillatory power spectra tend to flatten during the course of healthy ageing (Voytek et al., 2015;Merkin et al., 2023); conversely, the effect of neurological diseases can cause a steeper spectra, due to increasing low-frequency activity, and decreasing high-frequency activity. These mutually occurring changes are characterised as neural slowing, in which the amplitude across global power spectra shifts toward lower frequencies. Multiple methods for modelling neural slowing have been proposed, including utilising the coefficients of 1/f (Merkin et al., 2023;Voytek et al., 2015) and mean frequency (Fernández et al., 2006) of power spectra, as well as linear models of band limited power z-scored using a normative group (Wiesman et al., 2022).

Irrespective of methodology, neural slowing decreases with healthy ageing (Merkin et al., 2023;Voytek et al., 2015), which results from the same spectral trajectories of healthy ageing which we have demonstrated, namely the decreasing low-frequency (delta and theta) activity, and increasing high-frequency activity (beta and gamma), where this change in spectral activity pivots around and within the alpha band. However, individual and group-level deviations from the established spectral trajectory of age have been associated with the presence of neurodegenerative diseases. This has been established using both band limited power and spatially resolved neural slowing as a biomarker of AD (Chen et al., 2015;Giustiniani et al., 2022;Horvath et al., 2018;Ishii et al., 2017;Maestú & Fernández, 2020;Mandal et al., 2018;Wiesman et al., 2022) and Parkinson’s disease (Bočková & Rektor, 2019;Oswal et al., 2013;Vardy et al., 2011). Additionally, increasing delta activity has been shown to correlate with poorer cognitive performance in both healthy and mild cognitive impairment (MCI) participants (Cuesta et al., 2015), and decreasing alpha has been associated with worse cognitive scores along the control, MCI, mild AD continuum (Babiloni et al., 2006).

### 4.3 Maturational effects on functional coupling in intrinsic brain networks

Ageing also causes FC to change, though this is less comprehensively characterised than spectral power. Our findings suggest frequency-dependent changes across inter- and intra-network nodes throughout the brain. Specifically, a diffuse increase in inter-network connectivity across delta, beta, and gamma, as well as increasing visual network connectivity in alpha, beta, and gamma FC has been found to decrease in networks that contribute to higher cognitive functions such as the default mode, cingulo-opercular, and fronto-parietal control networks, as well as increase between networks such as the visual and somatomotor networks (Geerligs et al., 2015). As cognitive function declines with age, this aligns with the reduced DMN FC in delta, and increased visual-motor FC in delta and beta that we see in our analysis. Global FC has been shown to increase with age, prompted by increasing intra- and inter-network connectivity (Schäfer et al, 2014). Graph theoretic measures have also been applied to FC, with modularity and local efficiency being shown to decrease with respect to age (Geerligs et al., 2015).

The phase-locking value was found to be lower in alpha and beta frequency bands in MCI subjects compared to controls when at rest (López et al., 2014). Similarly, coherence and synchronisation likelihood are lower in MCI than in controls, with both measures being significant in the beta band (Gómez et al., 2009). A lower global FC has been witnessed in AD when compared to both subjective cognitive decline and control subjects, particularly in alpha and beta (Koelewijn et al., 2017;Schoonhoven et al., 2022), which alongside the specified increase in global FC with healthy ageing (Schäfer et al, 2014) again depicts the diverging connectivity of healthy and pathological trajectories, even when age is the greatest risk factor of the relevant disease. This example highlights the importance of comprehensive models of neurophysiological change with age, as those with AD would logically have “younger” looking brains based solely on these connectomic relationships.

### 4.4 Predicting brain age from neurophysiological data

Multiple studies have assessed the applicability of neuroimaging in brain age prediction, with most literature utilising T1 MRI data to test and train regression models. Structural imaging techniques have largely shown greater predictive power across regression metrics for this task, in part due to the greater sample sizes, but primarily due to the presence of clear anatomical effects of ageing. Decreasing brain volume, white and grey matter (Gunning-Dixon et al., 2009;Lockhart & DeCarli, 2014;MacDonald & Pike, 2021) alongside increasing ventricle and cerebral spinal fluid volume (MacDonald & Pike, 2021) give clear indications as to the age of the participant. Furthermore, associations between structural atrophy and cognitive decline are suggested, particularly with respect to cortical thinning rates (MacDonald & Pike, 2021) and white matter reductions (Gunning-Dixon et al., 2009). These trends further generalise to neurodegenerative diseases, in which similar regional dependent atrophy in grey and white matter is present (Young et al., 2020), and thus pathological neurodegeneration can appear in structural neuroimaging as an acceleration of the natural processes of ageing. This benefits the premise of predicted brain age as we naturally equate higher age to worse cognition.

Prediction of age via structural imaging of the brains of healthy subjects has been explored via a variety of algorithmic approaches (Cole et al., 2018;de Lange et al., 2022;Peng et al., 2021;Xifra-Porxas et al., 2021) which have achieved mean absolute error as low as 2.95 years (Peng et al., 2021). Given the success of this technique, research has also explored the relationship between brain age predictions/BD and various diseases. Brain age predictions on AD cohorts using structural imaging have shown higher BD compared to controls (Lee et al., 2022;Rokicki et al., 2021), as well as a progression of higher BD related to severity of neurodegeneration (Franke & Gaser, 2012;Lee et al., 2022) and cognitive impairment (Franke & Gaser, 2012;Poloni et al., 2022) within and across neurological diseases such as MCI, AD, preclinical AD, frontotemporal dementia, and dementia with Lewy bodies. Conversion between disease groups at a later stage has been related to BD at baseline (Franke & Gaser, 2012;Gaser et al., 2013;Lee et al., 2022). Higher BD has also been associated with ischemic heart disease (Rauseo et al., 2023), increased mortality risk (Cole et al., 2018), traumatic brain injury (Cole et al., 2015), and schizophrenia (Nenadić et al., 2017). However, it appears that higher BD does not generalise past schizophrenia to other psychiatric disorders (Nenadić et al., 2017) unless functional imaging is also utilised (Rokicki et al., 2021).

The benefit of multimodality in predicting brain age and relating BD to non-imaging data highlights that structural and functional imaging explain distinct features of importance with respect to ageing (Xifra-Porxas et al., 2021) and neurophysiological disorders (Cole, 2020;Niu et al., 2020). Age prediction via functional imaging is possible due to the prior mentioned changes in neural oscillations and FC, namely the flattening of the power spectra alongside increasing intra/inter FC. Furthermore, functional imaging may be preferential for disorders which do not present with structural abnormalities such as atrophy. FC via functional magnetic resonance imaging (fMRI) has been used to predict brain age (Li et al., 2018;Millar et al., 2022) and has shown the same relationship between BD and the severity of neurodegeneration across preclinical to symptomatic AD cohorts (Millar et al., 2022). Electrophysiological neural dynamics of the brain have also been used to predict participant age via EEG and MEG, both at rest (Al Zoubi et al., 2018;Engemann et al., 2022;Sabbagh et al., 2019,^2020^;Vandenbosch et al., 2019) and during sleep (Paixao et al., 2020;Sun et al., 2019;Ye et al., 2020), with mean absolute error in age prediction between 7.30 and 10.68 for MEG and between 6.48 and 10.78 for EEG when considering participants across the adult lifespan. This highlights that our predictive performance of age using neurophysiological data is inline with present research. MEG and EEG have also been used to demonstrate further replication of increased BD being linked to life expectancy, neurological and psychiatric disorders between clinical groups as compared to healthy controls (Paixao et al., 2020;Sun et al., 2019;Ye et al., 2020). However, the divergent changes in spectral activity and FC between healthy ageing and age-related pathological neurodegeneration elucidate the benefits of multimodality in this domain, as well as the need for further research into pathological neurophysiology through the lens of BD.

The relative importance of specific MEG features when predicting age has been measured, with spectral power proving more reliable than FC (Engemann et al., 2020;Xifra-Porxas et al., 2021), which we also determined during cross-validation using a variety of data subsets. Source power in the alpha and beta bands are noted to provide the most robust age-associated features, and furthermore add the most information to multimodality models, including other functional neuroimaging modalities (Engemann et al., 2020). Additionally, dimensionality reduction has been shown to improve performance and interpretability, particularly canonical correlation analysis, which is algorithmically similar to the PLS method we employed for exploration. However, standard dimensionality reduction techniques operating on only the independent features (such as PCA) tend to negatively affect predictive performance (Xifra-Porxas et al., 2021).

Although we only explored biophysical features, which benefit from ease of interpretation, it is unclear from the present literature whether this is the optimal data processing strategy for age prediction. Alternative approaches such as using Riemannian methods have shown both better and worse regression performance than biophysically inspired features, including on the same dataset (Engemann et al., 2022,Sabbagh et al., 2019), a difference which is associated with use of individual or average head models. This highlights how even limited differences in preprocessing can extrapolate into greater disparities in performance.

Age-dependent prediction bias is present in our regression results, with positive and negative BD for younger and older participants respectively. This phenomenon is present throughout the related literature (More et al., 2023;Peng et al., 2021;Smith et al., 2019) and we evaluated a method of bias correction explored in other studies, though this resulted in minimal bias reduction due to a large difference in fit between train and testing data (i.e., overfitting). Despite the limited effectiveness of the chosen correction method in this analysis, the application of bias correction did result in a decrease in mean absolute error for both train and test sets, highlighting the benefit of such techniques in this domain. However, correlation of bias-corrected BD to non-imaging data should be equivalent to including age as a covariate to statistically adjust by (de Lange & Cole, 2020), assuming optimal bias correction.

Given the established associations between neural oscillations/connectivity and age as measured by functional neuroimaging techniques such as EEG and MEG, and the ability to detect irregular/pathological activity, we hypothesise that functional neuroimaging-derived measures of “brain age” are sensitive to atypical activity, such as that witnessed within neurodegenerative cohorts.

## 5 Conclusion

Our findings suggest that strong associations exist between healthy ageing and neurophysiological features measured by MEG, such as spectral power and FC. In general, shifting spectral power from low to high frequencies across the whole brain, and decreasing intra- and inter-visual network connectivity were found to be correlated with healthy ageing. These associations were replicated using multiple independent techniques and were found to be spectrally and spatially consistent. Analysis of FC using MEG has previously been limited to cohorts composed of early childhood to midlife, here we expand into later life (18–83 years), extending the literature in this area. Cross-validated regression modelling was performed to predict participant age, with results showing mean absolute error of 8.54 years over mean intra-individual predictions.

## Supplementary Material

Supplementary Material

## Data Availability

Raw data are available from:
Open MEG Archive (OMEGA):https://www.mcgill.ca/bic/neuroinformatics/omega Donders Institute Mother of Unification Studies (MOUS):https://data.ru.nl/collections/di/dccn/DSC_3011020.09_236?0 National Institute of Mental Health (NIMH):https://openneuro.org/datasets/ds004215/versions/1.0.3 Code was used from:
Python:https://www.python.org/ Analysis:
MNE:https://mne.tools/stable/index.html numpy:https://numpy.org/ pandas:https://pandas.pydata.org/ pingouin:https://pingouin-stats.org/build/html/api.html scipy:https://scipy.org/ sklearn:https://scikit-learn.org/stable/index.html statsmodels:https://www.statsmodels.org/stable/index.html Visualisation:
matplotlib:https://matplotlib.org/ pyvista:https://docs.pyvista.org/version/stable/ seaborn:https://seaborn.pydata.org/ Freesurfer:https://surfer.nmr.mgh.harvard.edu/
