# Supplementary material for: Predicting brain age across the adult lifespan with spontaneous oscillations and functional coupling in resting brain networks captured with magnetoencephalography
Source: Imaging Neurosci (Camb). 2024 Jun 17;2:imag-2-00195. doi: 10.1162/imag_a_00195 (PMC12272185; doi:10.1162/imag_a_00195)
Supplement: Supplementary Material [file imag_a_00195-supp.pdf]

Supplementary Materials

Network Nodes Definitions

Central Executive Network

RIPS [25, -62, 53]
RVV [36, -62, 0]
LVV [-44, -60, -6]
RSMG [32, -38, 38]
RSLOC [26, -64, 54]
LSLOC [-26, -60, 52]
RFEF [28, -4, 58]
LFEF [-26, -8, 54]

Default Mode Network

LAG [-43, -76, 35]
RAG [51, -64, 32]
PCC [-3, -54, 31]
vMPFC [-2, 51, 2]
dMPFC [-13, 52, 23]
RMPFC [2, 53, 24]
LITG [-57, -25, -17]

Motor network

Precentral_L [-39.0, -7.0, 50.0]
Precentral_R [41.0, -10.0, 51.0]
Postcentral_L [-43.0, -24.0, 47.0]
Postcentral_R [41.0, -27.0, 51.0]
Parietal_Sup_L [-24.0, -61.0, 58.0]
Parietal_Sup_R [26.0, -60.0, 61.0]
Parietal_Inf_L [-43.0, -47.0, 45.0]
Parietal_Inf_R [46.0, -48.0, 48.0]

Attention network

RSMG [52, -48, 28]
RFEF [30, -13, 53]
LFEF [-26, -12, 53]
LpIPS [-25, -67, 48]
RpIPS [23, -69, 49]

LMT	[-43, -72, -8]
RMT	[42, -70, -11]
RMFG	[41, 17, 31]
RPCS	[41, 2, 50]
RSTG	[58, -48, 10]
RVFC	[40, 21, -4]

Visual network

LV1	[-3, -101, -1]
RV1	[11, -88, -4]
LV2d	[-8, -99, 7]
RV2d	[14, -96, 13]
LV3	[-9, -96, 13]
RV3	[20, -95, 18]
LV4	[-31, -77, -17]
RV4	[27, -71, -14]
LV7	[-23, -78, 26]
RV7	[32, -78, 25]

Abbreviations:

"RIPS": "Right Intra Parietal Sulcus",
"RVV": "Right Ventral Visual",
"LVV": "Left Ventral Visual",
"RSMG": "Right Supramarginal Gyrus",
"RSLOC": "Right Superior Lateral Occipital Cortex",
"LSLOC": "Left Superior Lateral Occipital Cortex",
"RFEF": "Right Frontal Eye Field",
"LFEF": "Left Frontal Eye Field",
"LAG": "Left Angular Gyrus",
"RAG": "Right Angular Gyrus",
"PCC": "Posterior Cingulate Cortex",
"vMPFC": "Ventromedial Prefrontal Cortex",
"dMPFC": "Dorsomedial Prefrontal Cortex",
"RMPFC": "Rostral Medial Prefrontal Cortex",
"LITG": "Left Inferior Temporal Gyrus",
"Precentral_L": "Precentral Left",
"Precentral_R": "Precentral Right",
"Postcentral_L": "Postcentral Left",
"Postcentral_R": "Postcentral Right",
"Parietal_Sup_L": "Parietal Superior Left",
"Parietal_Sup_R": "Parietal Superior Right",
"Parietal_Inf_L": "Parietal Inferior Left",
"Parietal_Inf_R": "Parietal Inferior Right",

"LV1": "Left Visual 1",
"RV1": "Right Visual 1",
"LV2d": "Left Visual 2 (Dorsal)",
"RV2d": "Right Visual 2 (Dorsal)",
"LV3": "Left Visual 3",
"RV3": "Right Visual 3",
"LV4": "Left Visual 4",
"RV4": "Right Visual 4",
"LV7": "Left Visual 7",
"RV7": "Right Visual 7",
"LpIPS": "Left posterior Intra-Parietal Sulcus",
"RpIPS": "Right posterior Intra-Parietal Sulcus",
"LMT": "Left Middle Temporal",
"RMT": "Right Middle Temporal",
"RMFG": "Right Middle Frontal Gyrus",
"RPCS": "Right Precentral Sulcus",
"RSTG": "Right Superior Temporal Gyrus",
"RVFC": "Right Ventro-Frontal Cortex"

Analysis Methodology

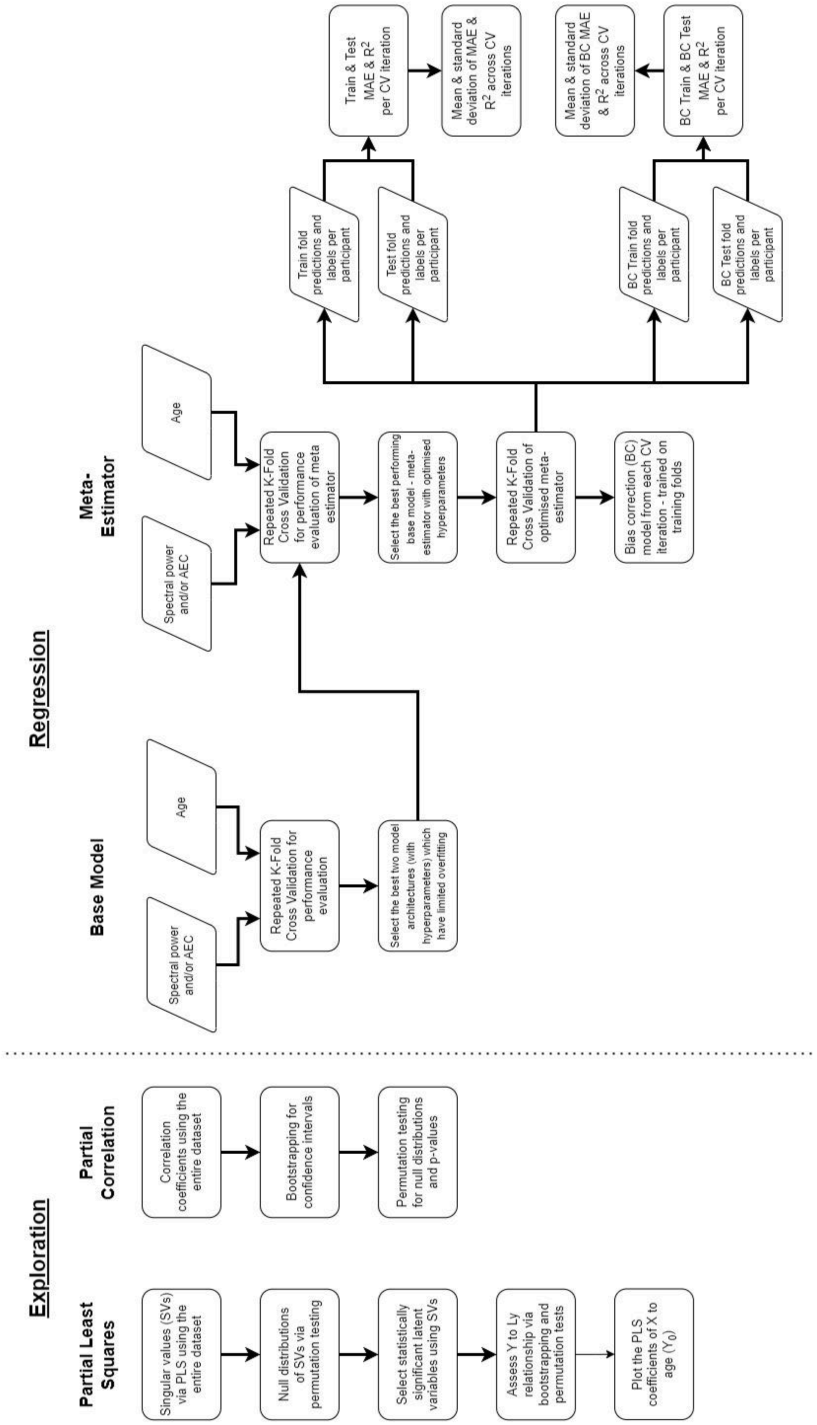

Figure S1: *A diagrammatic description of the data analysis methodology which was used in this work. The exploratory analysis steps for both partial correlation and partial least squares are detailed, alongside the two stages (base model and meta-estimator) of the regression analysis performed.*

Hyperparameter Tuning - Explored Parameters

RidgeCV

params = {"Model__alphas": [(1, 10, 100), (10, 100, 1000), (1000, 10000, 100000)]}

LassoCV

params = {"Model__eps": [(1e-1), (1e-2), (1e-3), (1e-4), (1e-5)],
"Model__alphas": [(1, 10, 100), (10, 100, 1000), (1000, 10000, 100000)]}

RandomForestRegressor & ExtraTreesRegressor

params = {"Model__min_samples_split": [2, 4, 8],
"Model__max_depth": [None, 4, 8, 16],
"Model__max_features": [1.0, "sqrt", "log2"]}

MLPRegressor

params = {"Model__hidden_layer_sizes": [(10), (500), (1000), (10, 20),
(500, 1000), (1000, 2000)],
"Model__alpha": [0.0001, 0.001, 0.01],
"Model__max_iter": [10000]}

SVR

params = {"Model__kernel": ["poly", "linear", "rbf"],
"Model__degree": [1, 2, 3],
"Model__gamma": ["auto", "scale"],
"Model__C": [0.0001, 0.001, 0.01, 0.1, 1, 10, 100],
"Model__epsilon": [0.1, 1.0, 10],
"Model__tol": [1e-7, 1e-6, 1e-5, 1e-4]}

AdaBoostRegressor + RidgeCV

params = {"Model__n_estimators": [1000],
"Model__learning_rate": [0.01, 0.1, 0.5, 1.0],
"Model__loss": ['linear', 'square', 'exponential'],
"Model__base_estimator__alphas": [(1, 10, 100), (10, 100, 1000), (1000, 10000, 100000)]}

AdaBoostRegressor + LassoCV

params = {"Model__n_estimators": [1000],
"Model__learning_rate": [0.01, 0.1, 0.5, 1.0],
"Model__loss": ['linear', 'square', 'exponential'],
"Model__base_estimator__eps": [(1e-1), (1e-2), (1e-3), (1e-4), (1e-5)],

"Model__base_estimtaor__alphas": [(1, 10, 100), (10, 100, 1000), (1000, 10000, 100000)]}

Partial Correlation

To assess the trends of spectral and connectivity features with respect to age we used partial correlation, which allows us to quantify the relationship between two continuous variables while removing the effects of any potentially confounding variables. In practice this allows us to confidently assess the effect of age on functional activity, while removing the effects of sex. In the case of multiple confounding variables, the partial correlation coefficient is calculated by first performing a multiple linear regression in which confounding variables are considered regressors alongside one of the continuous variables of the correlation, the second of the continuous variables is set as the response variable. Once regression has been performed, equation 1 is used to transform the regression results for a continuous variable into the partial correlation coefficient, where t_f is the student's t-test of the multiple regression coefficient β_f, p is the number of explanatory variables, and n is the number of samples.

$$r = \frac{t_f}{\sqrt{t^2_f + (n - p - 1)}} \quad (1)$$

Tests are performed by using one MEG derived feature, chronological age, and all relevant confounding variables (sex). Each of these tests was replicated on 1000 bootstrapped samples such that confidence intervals of the correlation coefficients can be calculated. Permutation tests were used to further assess the statistical significance of the tests, by generating a null distribution of the test statistic to calculate a p-value via comparison to the test statistic found using the full population.

The results of the partial correlation analysis show a general trend of oscillatory slowing associated with age, as delta and theta band power decreases, and beta and gamma activity increases. Spectral power across bands was generally more highly correlated and statistically significant than connectivity, which resulted in a higher proportion of the Bonferroni corrected results originating from the spectral features.

The results of the partial correlation analysis of spectral power show age dependent features of healthy ageing which are temporally and regionally specific, as well as highly bilateral. Delta power decreases across all but the occipital lobe with ageing (**Figure S2a**), similarly theta activity tends to decrease, with temporal and parietal regions presenting the strongest association with age (**Figure S2b**). Alpha spectral activity has the weakest associations with age, and is the only frequency band with bidirectional changes, with a subset of occipital and parietal regions showing decreasing power, and conversely temporal and frontal areas showing increasing activity (**Figure S2c**). Power in the beta band displays the strongest correlations with age (**Figure S2d**), with all regions proving statistically significant post multiple comparisons correction. Gamma spectral power continues the trend of oscillatory slowing, as positive correlations are present primarily in the occipital and frontal lobes (**Figure S2e**).

Across the canonical frequency bands of delta, theta, alpha, beta, and gamma, the left middle frontal gyrus orbital part ($r(367)=-.56$, $p=2.81e-24$), right precentral ($r(367)=-.56$, $p=1.27e-22$), right cuneus ($r(367)=-.36$, $p=3.84e-9$), left postcentral ($r(367)=.64$, $p=2.68e-29$), and right lingual ($r(367)=.48$, $p=7.26e-18$) regions had the greatest age association respectively.

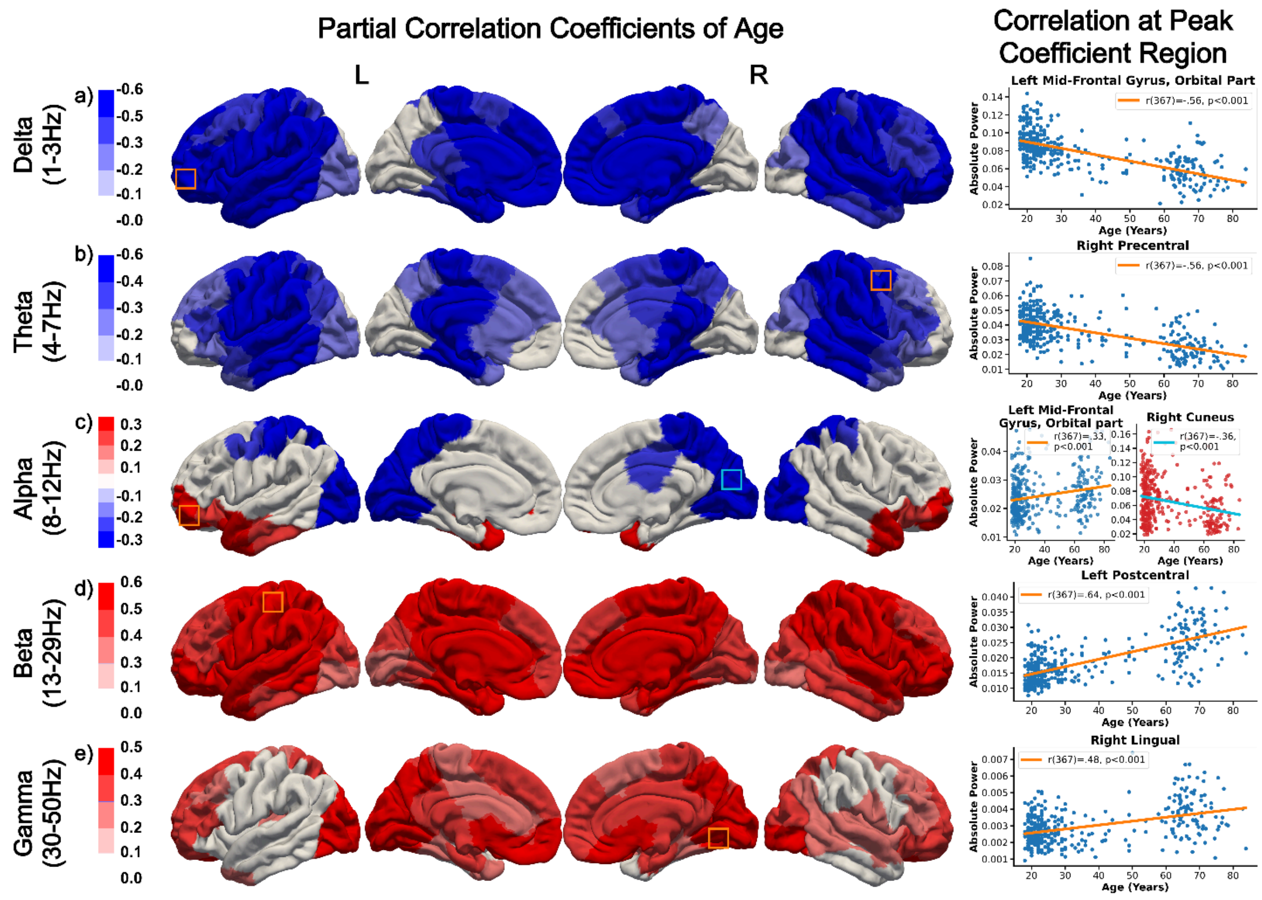

Figure S2: *Healthy ageing is associated with monotonic decreases in low frequency activity (e.g. delta and theta), and simultaneous increases in high frequency activity (e.g. beta and gamma), with regionally dependent bidirectional changes in alpha across prefrontal, temporal, and occipital locations. The strongest relationships between healthy ageing and spectral power were found in beta, which presents widespread increases across the brain to be associated with healthy ageing. These results show the Bonferroni corrected partial correlation coefficients of spectral power and healthy ageing. Bounding boxes on the band limited power plots highlight partial correlation coefficient peak regions, the colour of the bounding box corresponds to the line of best fit shown on the adjacent scatter plot, which shows data from this region.*

Decreased connectivity in the theta band was associated with age in a limited number of inter-network connections of the central executive, motor, and attention networks. Alpha connectivity likewise shows decreases with age, primarily in the visual network intra and inter network connections. Beta connectivity increased in a subset of default mode intra-network connections, and conversely beta and gamma connectivity decreased in subsets of visual intra-network connections.

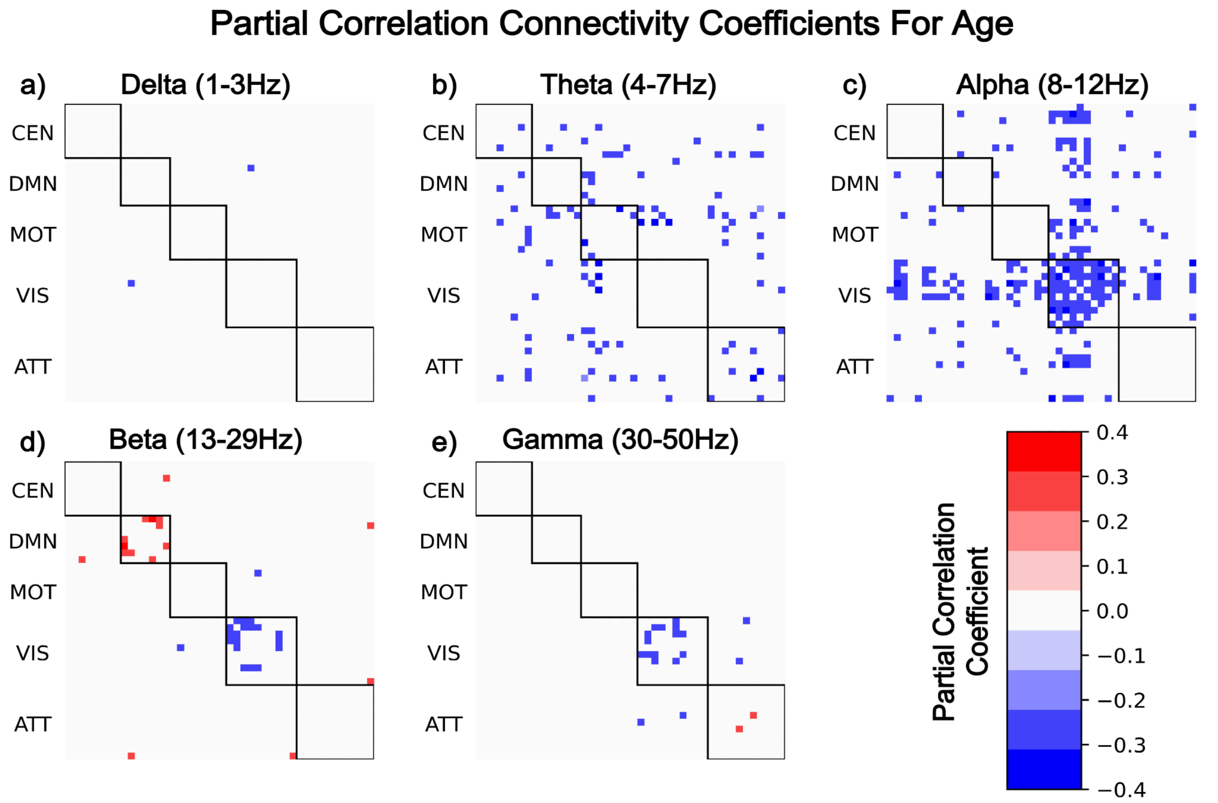

Figure S3: *Healthy ageing is associated with decreasing intra visual network connectivity in alpha, beta, and gamma, as well as decreasing inter visual network connectivity in alpha. Increasing default mode network connectivity in the beta band was found in a subset of connections. These results show the Bonferroni corrected partial correlation coefficients of functional connectivity and healthy ageing.*

To establish the degree of coherence between the PLS weights and partial correlation coefficients of the spectral power and FC features we used spearman's rank correlation. The results of this analysis are shown in **Table S1** and highlight that although associations between age and spectral power features were on average more consistent across different techniques than FC, the relationships between beta AEC and age were also highly stable. The lower correlation between PLS weights and partial correlation coefficients for AEC is potentially motivated by the lack of a clear age association in many inter-network connections. Alternatively, lack of correlation between these two measures could be the result of non-linear relationships between age and a given feature or set of features, as spearman's rank correlation used in partial correlation captures monotonic relationships rather than the linear association of PLS when considered at the non-latent variable level.

Table S1: *Correlation between PLS weights and partial correlation coefficients demonstrates the degree of coherence in the derived effects of healthy ageing by the two methods. Alpha and theta spectral power show the greatest correlation between methods, followed by beta functional connectivity, providing credence to the notion of beta connectivity being the most stable and implicated frequency band for connectivity throughout the brain.*

	Delta	Theta	Alpha	Beta	Gamma
Spectral Power (r, p)	.66, 3.77e-11	.93, 3.25e-35	.95, 2.60e-41	.79, 1.72e-18	.66, 3.39e-11
AEC (r, p)	.56, 3.85e-80	.45, 6.97e-50	.63, 2.23e-106	.84, 9.92e-254	.57, 8.60e-84
